# Long COVID: mechanisms, risk factors and recovery

**DOI:** 10.1113/EP090802

**Published:** 2022-11-22

**Authors:** Rónan Astin, Amitava Banerjee, Mark R. Baker, Melanie Dani, Elizabeth Ford, James H. Hull, Phang Boon Lim, Melitta McNarry, Karl Morten, Oliver O'Sullivan, Etheresia Pretorius, Betty Raman, Demetris S. Soteropoulos, Maxime Taquet, Catherine N. Hall

**Affiliations:** ^1^ Department of Respiratory Medicine University College London Hospitals NHS Foundation Trust London UK; ^2^ Centre for Human Health and Performance Institute for Sport Exercise and Health University College London London UK; ^3^ Institute of Health Informatics University College London London UK; ^4^ Department of Cardiology Barts Health NHS Trust London UK; ^5^ Faculty of Medical Sciences Newcastle University Newcastle upon Tyne UK; ^6^ Imperial Syncope Unit Imperial College Healthcare NHS Trust London UK; ^7^ Brighton and Sussex Medical School Falmer UK; ^8^ Institute of Sport Exercise and Health (ISEH) Division of Surgery and Interventional Science University College London London UK; ^9^ Royal Brompton Hospital London UK; ^10^ Applied Sports, Technology, Exercise and Medicine Research Centre Swansea University Swansea UK; ^11^ Nuffield Department of Women's and Reproductive Health University of Oxford Oxford UK; ^12^ Academic Department of Military Rehabilitation Defence Medical Rehabilitation Centre Stanford Hall Loughborough UK; ^13^ School of Medicine University of Nottingham Nottingham UK; ^14^ Department of Physiological Sciences Faculty of Science Stellenbosch University Stellenbosch South Africa; ^15^ Department of Biochemistry and Systems Biology Institute of Systems Molecular and Integrative Biology Faculty of Health and Life Sciences University of Liverpool Liverpool UK; ^16^ Radcliffe Department of Medicine Division of Cardiovascular Medicine University of Oxford Oxford UK; ^17^ Radcliffe Department of Medicine Division of Cardiovascular Medicine Oxford University Hospitals NHS Foundation Trust Oxford UK; ^18^ Department of Psychiatry University of Oxford Oxford UK; ^19^ Oxford Health NHS Foundation Trust Oxford UK; ^20^ School of Psychology and Sussex Neuroscience University of Sussex Falmer UK

**Keywords:** cardiovascular, coagulation, dysautonomia, fatigue, long COVID, ME/CFS, respiratory, SARS‐CoV‐2

## Abstract

Long COVID, the prolonged illness and fatigue suffered by a small proportion of those infected with SARS‐CoV‐2, is placing an increasing burden on individuals and society. A Physiological Society virtual meeting in February 2022 brought clinicians and researchers together to discuss the current understanding of long COVID mechanisms, risk factors and recovery. This review highlights the themes arising from that meeting. It considers the nature of long COVID, exploring its links with other post‐viral illnesses such as myalgic encephalomyelitis/chronic fatigue syndrome, and highlights how long COVID research can help us better support those suffering from all post‐viral syndromes. Long COVID research started particularly swiftly in populations routinely monitoring their physical performance – namely the military and elite athletes. The review highlights how the high degree of diagnosis, intervention and monitoring of success in these active populations can suggest management strategies for the wider population. We then consider how a key component of performance monitoring in active populations, cardiopulmonary exercise training, has revealed long COVID‐related changes in physiology – including alterations in peripheral muscle function, ventilatory inefficiency and autonomic dysfunction. The nature and impact of dysautonomia are further discussed in relation to postural orthostatic tachycardia syndrome, fatigue and treatment strategies that aim to combat sympathetic overactivation by stimulating the vagus nerve. We then interrogate the mechanisms that underlie long COVID symptoms, with a focus on impaired oxygen delivery due to micro‐clotting and disruption of cellular energy metabolism, before considering treatment strategies that indirectly or directly tackle these mechanisms. These include remote inspiratory muscle training and integrated care pathways that combine rehabilitation and drug interventions with research into long COVID healthcare access across different populations. Overall, this review showcases how physiological research reveals the changes that occur in long COVID and how different therapeutic strategies are being developed and tested to combat this condition.

## Abstract (highlights)

- **What is the topic of this review?**
  The emerging condition of long COVID, its epidemiology, pathophysiological impacts on patients of different backgrounds, physiological mechanisms emerging as explanations of the condition, and treatment strategies being trialled. The review leads from a Physiological Society online conference on this topic.
- **What advances does it highlight?**
  Progress in understanding the pathophysiology and cellular mechanisms underlying Long COVID and potential therapeutic and management strategies.

## 1 INTRODUCTION

Long COVID, persistent post‐COVID symptoms of 12 weeks or more, threatens individuals, populations and economies, with an estimated 1.4 million individuals affected in the UK alone, and 144 million globally (Ayoubkhani & Pawelek, 2022; Wulf Hanson et al., 2022). The problem of post‐viral syndromes is not new, but the scale and the speed of this global challenge necessitate joined‐up thinking across traditional clinical and academic silos. As long COVID is an emerging condition, its epidemiology, risk factors and mechanisms must be identified in order to understand its aetiology, aid diagnosis and inform novel treatment strategies, which themselves need to be assessed while being made accessible to an ever‐increasing patient cohort. New research and ongoing investigations and treatment trials are critical for combatting a condition with such significant impacts on population and individual health. This paper reflects many perspectives from a recent Physiological Society meeting (‘Long COVID: Mechanisms, Risk Factors and Recovery’), which brought together diverse research groups tackling long COVID, to enable a greater understanding of the condition and identify directions for future research and treatment.

## 2 THE IMPORTANCE OF DATA IN CLASSIFYING LONG COVID

It is vital that long COVID is consistently described as a condition and that prevalence rates are accurately assessed, but neither of these are straightforward. Long COVID study design and recruitment can dramatically affect estimates of prevalence, reported symptoms and their duration, and associated risk factors. Additional factors to consider are including testing status, presentation and duration. Measuring the prevalence and incidence of long COVID is therefore fraught with difficulty, though vital if the impact on individuals and society is to be adequately addressed.

In the early days of the pandemic, many people experiencing community‐managed COVID‐19 could not access COVID‐19 testing and could not be recruited to studies. Therefore, long COVID rates were initially estimated in patients who had been hospitalised – in which post‐intensive care unit illness and disability was expected (up to 87%) (Carfì et al., 2020). Later, due to patient pressure, long COVID in non‐hospitalised populations started to be recognised (Assaf et al., 2020).

Diagnosis from symptom clusters has been challenging, as long COVID symptoms (fatigue, headache, brain fog, musculoskeletal pain, shortness of breath (SoB), chest pain) are common in the general population (Kirmayer et al., 2004). Non‐controlled studies cannot therefore readily discriminate levels and severity of symptoms between those with or without exposure to COVID‐19. This is compounded by pandemic responses such as lockdowns and school closures, which resulted in increased stress levels, social isolation, modified lifestyle habits, reduced physical activity, relationship difficulties, and social and economic insecurity for many people, independently associated with increased physical symptoms (Kirmayer et al., 2004). Additionally, without pre‐illness participant baseline data, the impact of COVID‐19 on pre‐existing symptoms cannot be determined.

There is no commonly understood definition for symptom duration, with studies measuring between 3 and 24 weeks – with such heterogeneity precluding meta‐analyses (Cabrera Martimbianco et al., 2021). Further complications exist with the variable vaccination status and predominant COVID‐19 variant over time.

Studies recruiting patients with self‐reported long COVID can describe the characteristics and impact (Ziauddeen et al., 2022), organ damage and physiological changes (Dennis et al., 2021), but cannot estimate of prevalence or duration, due to self‐selection bias. Retrospective (post‐COVID‐19 infection) cohorts help estimate prevalence, but may over‐estimate the impact of COVID‐19 on chronic symptoms without baseline or comparison groups (Fernández‐de‐Las‐Peñas et al., 2021).

The strongest study design for understanding prevalence and duration of long COVID is a prospective cohort, ideally with pre‐illness baseline data, and an equivalent non‐exposed comparison group. The COVID‐19 symptom tracker app offers this (Sudre et al., 2021) – people who log data generally do so before their COVID‐19 infection, and once infected, can be compared to similar loggers who did not have COVID‐19. Other cohort resources are the UK Biobank (Griffanti et al., 2021), or population‐based electronic health records (Walker et al., 2021), although the latter is limited by requiring a consultation with a healthcare professional and correct coding by that professional.

## 3 PREVALENCE, NATURE AND DIFFERENCE FROM OTHER POST‐VIRAL ILLNESS

Given the above challenges, retrospective cohort studies based on routinely collected data extracted from electronic health records (EHR) can help. Such studies do not rely on patients self‐reporting their symptoms, nor on people self‐enrolling in studies. They can also provide baseline data for each patient, as well as control groups.

EHR data from over 81 million individuals, mostly in the USA, including 273,618 confirmed COVID‐19 patients, estimated the incidence of nine long COVID features 3–6 months after COVID‐19 versus influenza (Taquet et al., 2021). The incidence of any one feature in the COVID‐19 cohort was 42.34%, with prevalence of each symptom detailed in Table 1. All nine features were more frequently reported after COVID‐19 than after influenza (overall excess incidence 16.60%, hazard ratio range 1.44–2.04, all *P* < 0.001), with symptom co‐existence significantly more likely than after influenza. When long COVID features were represented as a network of symptoms (each node is a symptom, each edge represents their likelihood to co‐occur), this network was found to be more densely connected as time from COVID‐19 infection increased. In other words, when a post‐acute symptom was recorded, it was more likely to co‐occur in a constellation with other symptoms (compared to acute phase). Notably, 30% of post‐influenza individuals had at least one feature recorded 3–6 months post‐infection. While this was significantly less than after COVID‐19, this is not a trivial incidence and might indicate that part of the burden of long COVID might be attributed to a generic post‐infection illness.

**TABLE 1:** Proportion of symptoms experienced following acute COVID‐19 at 1–180 and 90–180 days post‐illness (Taquet et al., 2021)

	Proportion of symptoms (%)	
Symptom	1−180 days	90−180 days
Anxiety/depression	22.82	15.49
Abnormal breathing	18.71	7.94
Abdominal symptoms	15.58	8.29
Fatigue/malaise	12.82	5.87
Chest/throat pain	12.60	5.71
Other pain	11.60	7.19
Headache	8.67	4.63
Cognitive symptoms	7.88	3.95
Myalgia	3.24	1.54

Differences in long COVID features between different patient subgroups was also seen. Female patients were more likely to have headaches, myalgia and abdominal symptoms, and less likely to have abnormal breathing and cognitive deficits than male patients. Hospitalised patients and older individuals were more likely to have cognitive problems, abnormal breathing and fatigue, and less likely to have headaches and myalgia than non‐hospitalised patients or younger individuals.

Another study by the same group using the same EHR methodology (Taquet et al., 2022) found that the risk of developing long COVID features was overall very similar in those with and without a COVID‐19 vaccination. Vaccination did not reduce risk of anxiety/depression, headache, abdominal symptoms, chest/throat pain, abnormal breathing and cognitive symptoms. However, certain symptoms, notably fatigue and myalgia, were less common in the vaccinated population.

## 4 LINKS WITH ME/CFS

The increased incidence of symptoms after COVID‐19 compared to influenza suggests some specificity for the type of infection. However, there are also some similarities between long COVID and myalgic encephalomyelitis/chronic fatigue syndrome (ME/CFS). Like long COVID, this is associated with prior viral infection and often occurs in previously healthy and active people (predominantly females) (Poenaru et al., 2021). The chronic presentation of both conditions is similar, with fatigue, brain fog and post‐exertional malaise (PEM) (Singh et al., 2022), impacting on activities of daily living. One study demonstrated that in those symptomatic with long COVID at 2 months, 85% still reported symptoms after 1 year (Tran et al., 2022). Long COVID can have a significant impact on patients’ lives after 6 months, which may, in some cases, represent evolution to an ME/CFS‐like condition.

Life‐altering fatigue is very common in both populations and also in patients with chronic autoimmune (Davies et al., 2021) or neurological (Kluger et al., 2013) diseases. In some, this fatigue becomes severely disabling (van Ruitenbeek et al., 2019). Patients with ME/CFS need daily management of activity levels in order to prevent PEM, which when severe, can lead individuals to become bed‐bound, with symptoms such as severe postural orthostatic intolerance (POTS), sleep dysfunction, myalgia, cognitive dysfunction, dysautonomia, neuro‐immuno‐endocrine dysfunction, hyperacusis and photophobia (Carruthers et al., 2011; Stussman et al., 2020).

Similar to long COVID, the lack of a diagnostic test is a significant hurdle, with many patients diagnosed by excluding other conditions, or given a diagnosis of ME/CFS after a prolonged period of time (>10 years). Between 80% and 90% of patients never get a clear diagnosis (Komaroff et al., 1996). Current ME/CFS management options are limited, with graded exercise therapy removed in 2021 from NICE guidance as a result of 50% of patients experiencing a deterioration of their conditions (Kujawski et al., 2020, 2021; NICE Guideline [NG206], 2021). Treatments for depression or other psychiatric illness have limited benefit.

Once ME/CFS is established, many patients never fully recover and are ‘forgotten’ by society. However, the burden on families is enormous, with many families taking on a life‐long commitment as carers. A better understanding of long COVID, a prolonged condition with many of the same symptoms observed in ME/CFS, therefore presents an opportunity that may also help in understanding and managing patients with ME/CFS, with options for the development of therapeutic interventions in a potentially more homogeneous condition.

ME/CFS research can also help with understanding of underlying pathophysiological mechanisms, such as dysregulated energy metabolism (Missailidis et al., 2020; Sweetman et al., 2020; Tomas et al., 2017, 2020), exercise‐induced plasma metabolome alterations (Germain et al., 2022), dysbiotic gut (Morten et al., 2018; Xiong et al., 2021) and immune cell dysfunction (Milivojevic et al., 2020). Evidence of metabolic dysregulation and prolonged immune dysregulation has also been found in long COVID patients (Phetsouphanh et al., 2022). This could potentially be due to viral persistence of SARS‐CoV‐2 or other viruses, though this has not been demonstrated in ME/CFS patients (Chang et al., 2021). SARS‐CoV‐2 persistence of 3–5 months has been reported in immunocompromised patients, but with no co‐existing symptoms (Gaspar‐Rodríguez et al., 2021), and this is an area of further study (Brodin et al., 2022).

To progress, lessons must be learnt from the poor management of ME/CFS patients and long term research programmes established to fully understand the biology behind long COVID and ME/CFS. Certain insights may arise sooner in populations that have undergone particularly high levels of scrutiny of the disease course, progression and effects of interventions. These include individuals in elite athletics or the military, where physical fitness is key to success and resource‐intensive monitoring of performance is routine.

## 5 LONG COVID IN PHYSICALLY ACTIVE POPULATIONS

Several epidemiological studies have demonstrated that being physically inactive (performing below WHO recommendations for regular weekly physical activity) is associated with a 30% increase in hospitalisation risk (Hamer et al., 2020; Sallis et al., 2021), similar to having poorly controlled diabetes. Given these findings, it is unsurprising that athletic individuals, free from chronic illnesses, typically only develop mild acute symptomatology and rarely require hospital‐based care. However, even professional or international‐level athletes can struggle with protracted symptoms, often scuppering their competition participation in events such as the Tokyo Olympic Games (Falkingham, BBC, 2020). In another highly active population, military personnel, it was also clear early in the pandemic that post‐COVID‐19 pathology would impact on the role and deployability of the UK Armed Forces, although the degree of this was unknown. The degree of monitoring of physical health, and support structures for training and rehabilitation that exist for competitive athletes and the military provide the potential for unique insights into the nature, pathophysiology and potential treatment regimes for long COVID sufferers.

The questionnaire‐based AWARE I study in South African athletes showed that acute and post‐acute COVID pathology follows a similar pattern in active individuals to the general population (*n* = 45) (Schwellnus et al., 2021). Acute illness lasted longer than for other causes of respiratory tract infections, and a cluster of seven symptoms (‘excessive fatigue’, ‘chills’, ‘fever’, ‘headache’, ‘altered/loss sense of smell’, ‘chest pain/pressure’, ‘difficulty in breathing’ and ‘loss of appetite’) were associated with a more protracted return to sport at 40 days. Similar prolonged symptoms were reported by military personnel, including fatigue, cough, SoB and mood disturbance and were more common in those over 40 years of age (Holdsworth et al., 2022; O'Sullivan et al., 2021).

Prevalence of prolonged symptoms is also similar to the general population, with approximately 10% of elite athletes preparing for international‐level competition experiencing symptoms for >28 days (*n* = 147) (Hull, Wootten, Moghal et al., 2022; Sudre et al., 2021), with 27% experiencing a delay in full return to sport after 1 month and 6% still impacted at 90 days. This figure was substantially higher than pre‐COVID data, in which only 4% of athletes had not returned a month after respiratory tract illness. A delayed recovery was twice as common in athletes with a symptom presentation involving the lower respiratory tract (e.g., including SoB ± chest pain). More recent data from US collegiate‐based athletes (*n* = 3597), revealed a far lower prevalence of protracted symptoms, at only 0.8% at 28 days (Petek et al., 2021). Military personnel too showed a reduction prevalence of long COVID symptoms over time, with incidence reducing between pre‐immunisation wave one (wild‐type) and wave two (alpha variant), likely as a result of increased awareness of the condition, improved availability of self‐management strategies and the predominant SARS‐CoV‐2 variant (O'Sullivan, 2022; O'Sullivan et al., 2021). Further work is planned in the same population of UK Armed Forces to understand the effects of immunisation, and the delta and omicron variants of SARS‐CoV‐2. Vaccination itself is well‐tolerated in athletes, with very few reporting an impact of vaccination on training (Hull, Wootten, & Wootten, 2022).

Developing intervention strategies potentially helped reduce the prevalence of prolonged symptoms, and as seen in other post‐viral conditions, the NICE‐recommended multi‐modal and multi‐disciplinary team (MDT) approach offers holistic patient benefit in the management of long Covid (NICE guidelines, 2020). Within the UK Armed Forces, after recognising the need early in the pandemic, various pathways were created simultaneously, including remote rehabilitation assessment and subsequent rehabilitation programmes, a combined occupational and medical assessment clinic and a longitudinal observational study (Barker‐Davies et al., 2020; O'Sullivan, 2021; O'Sullivan et al., 2021; O'Sullivan, Barker‐Davies, Gough et al., 2021). The key question underpinning these was: How do we return a physically active population to full health and activity, at scale, without risk?

Data from these pathways suggest that approximately 2% of military personnel developed problems following COVID‐19, and two‐thirds of those required MDT rehabilitation, including education, pulmonary rehabilitation, symptom‐titrated activity progression, psychological assessment and input, and occupational support. These were delivered by self‐management and residential programmes, with a subsequent remote supported self‐directed programme. Three months after these interventions, 91% of individuals who received them were back in work, some with persistent symptoms, and nearly all still using strategies they were taught. Patients were armed with a self‐management educational booklet, linking into NHS resources such as Your COVID recovery. Over the course of the pandemic, these interventions became more community based, supported by GPs and allied health professionals, with specialist input reserved for severe or complex cases.

Cardiopulmonary exercise testing (CPET) was used as a cornerstone to understand the degree and nature of any limitation, recognising that most post‐COVID‐19 symptomology was related to, or worsened by, exertion (Holdsworth et al., in press, BMJ Military Health). CPET was used as part of a detailed clinical assessment, which also included blood tests, ECG, spirometry, cognitive assessment and 6‐min walk tests, with secondary care computed tomography/magnetic resonance imaging (MRI) if required (O'Sullivan et al., 2021). Reassuringly, extremely low levels of cardiopulmonary pathology were identified, alleviating concerns regarding occult organ pathology (Holdsworth et al., 2022), and paralleling data from athletes. Initially, the early‐cited high prevalence of myocarditis (i.e. circa 25–50%) delayed return to sport (Wilson et al., 2020), but these fears were not substantiated, with later research indicating the prevalence of clinical myocardial events is ≤1% in athletes (van Hattum et al., 2021).

Studies in the military population also helped identify potential contributing factors to long COVID including objective cognitive disturbance equivalent to ageing 10 years or exceeding the UK drink‐drive limit, exercise related dysautonomia in up to 25% of individuals, and exercise‐induced ventilatory inefficiency (Holdsworth et al., 2022; Ladlow et al., 2022). Similar changes were also found in athletes following COVID‐19, with alterations in both cardiovascular and respiratory response parameters impacting cardiorespiratory performance during CPET (Fikenzer et al., 2021). Athletes with long COVID also show symptoms of dysautonomia and ventilatory inefficiency, reporting perturbations in both their resting heart rate (HR) and cardiac response to sub‐maximal exertion as well as ‘unsatisfied respiration’ and seemingly disproportionate SoB. Several of these clinical features appear similar to the condition of unexplained underperformance or the over‐training syndrome, for example, fatigue and lack of recovery (Lewis et al., 2015). The precise pathobiological factors underpinning this syndrome remain elusive, but the commonality of this pathophysiology with long COVID warrants further investigation.

To understand the medium‐long term impact of COVID‐19 on the UK Armed Forces, the longitudinal cohort study ‘Military COVID‐19, Observational outcomes in a Viral Infectious Disease (M‐COVID)’ (1061/MODREC/20) was established. Initial findings demonstrate that fully recovered community‐based individuals are no different from a control population on any parameter 5 months post‐illness, but this is not the case for those who have recovered following hospital‐based illness (Ladlow, O'Sullivan, Bennett et al., 2022; O'Sullivan et al., 2022). These results suggest that those with initially severe or persistent symptoms should undergo appropriate investigations, such as CPET, prior to a safe return to strenuous physical activity.

Overall, evidence in this population of physically active individuals suggests a similar presentation and proportion of long COVID to other populations, despite, on the whole, much less severe initial illness. Individuals who have had a mild to moderate illness who appear fully recovered are indeed most likely recovered, allowing resources to be dedicated to those with prolonged or initially very severe illness. MDT rehabilitation, either remote or residential, can provide benefit for most individuals, with detailed assessment, including CPET, helpful to identify specific limitations.

## 6 WHAT DOES CARDIOPULMONARY EXERCISE TESTING TELL US?

As discussed above, CPET is a valuable tool to understand the limitations to individuals’ performance and thus the nature of required rehabilitation, but it is also helpful as a research tool to understand the various physiological processes that become dysfunctional in long COVID.

A siloed approach to understanding long COVID by individual medical science specialities is unlikely to provide unifying understanding of the underlying pathological mechanisms. As seen above, exertional limitation and fatigue are both prominent in the described symptomatology (Heightman et al., 2021) and their investigation demands an integrated whole‐body physiological approach. CPET is a robust, validated and evidenced method of assessing the multi‐system response to exercise, and as such has been identified as a means by which to unpick the mechanism(s) underlying long COVID.

However, the published evidence is limited by the majority of studies involving post‐hospital patient cohorts. These cohorts vary significantly from a non‐hospitalised long COVID group (Heightman et al., 2021) and exercise limitation in the setting of a more severe acute infection might be expected (Herridge et al., 2003, Puthucheary et al., 2013) independent of COVID‐19 specific pathology. However, it might be argued that SARS‐COV‐2 pathology significant enough to cause long lasting pathology in a ‘mild illness’ population should also be evident in those with more severe initial disease. As such, useful information might be gleaned from studies performed in the latter.

Rinaldo and colleagues studied a group 3 months after hospital discharge (Rinaldo, Mondoni, Parazzini, Pitari, et al., 2021). There was no apparent ventilatory limitation, though 55% had a reduced peak oxygen consumption (${\dot{V}}_{O_{2}}$) in a convincingly maximal‐testing protocol. The authors concluded that the pattern of early anaerobic threshold, blunted oxygen pulse and reduced ${\dot{V}}_{O_{2}}$/work rate was due to deconditioning. However, deconditioning is an imprecise diagnosis – in common practice, it suggests a passive process of muscle inefficiency, often secondary to decreased use (e.g., immobility, illness). With no direct measure of cardiac output or peripheral oxygen uptake, one cannot, however, exclude the possibility of reduced oxygen delivery or a more active aetiology of peripheral muscle pathology. Support for a peripheral muscle dysfunction has been demonstrated at hospital discharge (Baratto et al., 2021), by using exercise echocardiogram, arterial blood sampling and resolving the Fick equation to measure cardiac output and peripheral oxygen extraction. Peak ${\dot{V}}_{O_{2}}$ correlated with disease severity, and reductions in peak ${\dot{V}}_{O_{2}}$ correlated with reduced peripheral oxygen uptake, localising the pathology to the peripheral locomotor muscle. In this study, impaired ventilatory efficiency was also noted, a combination of raised respiratory rate and dead space ventilation, and increased chemosensitivity, a finding also noted by others at 3 months (Cassar et al., 2021; Skjørten et al., 2021).

Exercise limitation persists in the post‐hospital cohort, with around 20% of individuals still demonstrating peak ${\dot{V}}_{O_{2}}$ below 85% of their predicted maximum at 6 months (Cassar et al., 2021). Mounting evidence confirms a similar limitation in community‐managed long COVID (de Boer et al., 2022; Ladlow et al., 2022; Singh et al., 2022). Though small (*n* = 10, with matched controls), the study in still‐symptomatic community‐treated individuals at 12 months by Singh and colleagues advances understanding significantly. Using an invasive CPET protocol, the investigators directly measured cardiac output, oxygen delivery and extraction. In patients, beyond 75% of peak ${\dot{V}}_{O_{2}}$, exercise capacity was limited by failure to increase oxygen extraction in the periphery. De Boer and colleagues have suggested this is due to mitochondrial dysfunction, inferred by an apparent reduction in fatty acid oxidation during exercise (de Boer et al., 2022), but the control data in this study are historic and poorly matched, preventing firm conclusions at this point. However, given the evidence for direct and indirect mechanisms of mitochondrial impairment in COVID‐19 (Cortese et al., 2020; Flynn et al., 2021; Mao et al., 2020), this hypothesis appears worthy of further investigation.

Finally, these studies, as in other populations discussed above, also note autonomic dysfunction. Reduction in peak HR (Clavario et al., 2021), chronotropic incompetence (Jimeno‐Almazán et al., 2021) and dysautonomia (Ladlow et al., 2022) have all been documented in long COVID sufferers, though not always associated with the same pattern of peripheral muscle CPET limitation as described above, suggesting dysautonomia might an additional mechanism of limitation.

These data from CPET studies point us toward a peripheral muscle mechanism of exercise dysfunction in a significant cohort of individuals. Understanding this mechanism more fully with deeper phenotyping might improve understanding of other symptoms of this debilitating condition. However, it seems multiple processes are at play, including ventilatory inefficiency, and autonomic dysfunction. Identifying the presence and interplay of these mechanisms in an individual is likely to be important in both understanding their symptom set and personalising targeted therapy.

## 7 DYSAUTONOMIA AND POTS

As articulated previously, dysautonomia appears to be a feature of long COVID in several populations, often including alterations in HR regulation. Syncope and autonomic clinics globally, and including those of two of the authors (M.D., P.B.L.) have been reporting cases of cardiovascular autonomic disorders and peripheral autonomic neuropathy, both during and following Covid infection (Abrams et al., 2022; Blitshteyn & Whitelaw, 2021; Hinduja et al., 2021; Johansson et al., 2021; Shouman et al., 2021). A typical post‐COVID presentation involves SoB, palpitations and dizziness, markedly worse on standing (orthostasis). This includes postural orthostatic tachycardia syndrome (POTS), a condition characterised by an increase in HR >30 bpm on standing, without a drop in blood pressure (BP), accompanied by the above symptoms (Sheldon et al., 2015).

We systematically examined the head‐up tilt responses of 27 individuals who were referred with long Covid autonomic dysfunction. Distinct patterns of haemodynamic responses to standing were observed:

1. POTS – 15% of the cohort
2. POTS, baseline low or normal BP – 4% of the cohort
3. Sub‐threshold POTS (sustained HR rise <30 bpm but more than 15% rise from baseline), with baseline hypertension: 33% of the cohort
4. Sub‐threshold POTS, baseline low or normal BP: 30% of the cohort
5. Low baseline BP (systolic BP <100 mmHg in supine position), no significant HR or BP rise – 4% of the cohort
6. Within normal limits: 15% of the cohort

Markers of increased sympathetic nervous system activity were evident, with 85% of patients showing HR rise over more than 15% baseline on standing, and 30% of patients with baseline BP more than 130/80 mmHg. Most (78%) had BP oscillations on standing (variations in BP with peak to trough systolic BP over >30 mmHg over 120 s), a predictor of impending vasovagal syncope (Hausenloy et al., 2009) suggesting haemodynamic instability (Julu et al., 2003; Samniah et al., 2004).

These findings support those of other groups reporting varying rates of POTS from 22% to 75% (Blitshteyn & Whitelaw, 2021; Shouman et al., 2021). Unlike other groups, however (Buoite Stella et al., 2022; Shouman et al., 2021), no patients in this cohort had orthostatic hypotension, although 22% had systolic BP <100 mmHg during the supine phase. This may be due to referral bias to the cardiology‐led service, or due to the small numbers involved in this study.

While these findings represent a small cohort of patients who have been referred specifically for tilt table testing, the sympathetic overactivity is compelling, with most individuals experiencing HR and BP rise on standing. This suggests autonomic dysregulation – either newly incident following the infection or unmasked by the infection.

Commonly used medications for cardiovascular autonomic disorders such as POTS can include fluid expanders (fludrocortisone) and α‐agonists (midodrine) to augment BP, improving cardiac venous return and thus reducing overshoot sympathetic response. Additionally, β‐receptor blockers can mitigate symptoms from exaggerated sympathetic and adrenergic responses to orthostasis. The findings from this small cohort suggest that the former medications may not be indicated for this subgroup, and β‐blockers may be more helpful. Additionally, these patients are likely to benefit from interventions modulating the autonomic nervous system which specifically reduce sympathetic activity and increase vagal tone – such as breath retraining, HR variability (HRV) biofeedback training, and paced postural exercises incorporating breathing such as yoga.

## 8 NEUROLOGICAL CONTRIBUTIONS TO LONG COVID

The occurrence of dysautonomia and brain fog suggests neurological contributions to long COVID, and fatigue, one of the most common post‐COVID symptoms, could also be impacted by neurological dysfunction. To interrogate this, neuronal circuit dysfunction was investigated in patients suffering from fatigue at least 6 weeks following a mild or moderate COVID‐19 infection.

Inflammation is likely to be critical in the pathogenesis of post COVID‐19 sequelae. Individuals with long COVID have elevated inflammatory markers for several months (Phetsouphanh et al., 2022). There are multiple physiological pathways by which the immune system could influence the nervous system and vice versa (Dantzer, 2018). Whatever the molecular trigger, neuromuscular mechanisms must inevitably contribute to symptomatic fatigue, given that most common symptoms of post‐COVID fatigue relate to physical and cognitive activity, both of which rely on neural circuitry; however, which neural systems are affected is not known.

Volunteers with self‐reported post‐COVID fatigue underwent a battery of behavioural and neurophysiological tests assessing the central, peripheral and autonomic nervous systems (Baker et al., 2022). In comparison to age and sex matched volunteers without fatigue, differences in specific neural circuits were seen: primary motor cortex (M1), one of the most important areas for voluntary movements and driving muscles into action, was less excitable in the fatigued cohort. Fatigued individuals also had a higher HR and reduced HRV, both phenomena associated with dysautonomia, which is often associated with fatigue. Sensory feedback circuits and descending neuromodulatory control dysregulation were not impacted. Finally, myopathic changes in skeletal muscle were observed: although the fatigued cohort had normal levels of strength, after a sustained contraction, the ability of the muscle to generate force was reduced compared to controls.

These abnormalities on objective tests may indicate novel avenues for principled therapeutic intervention and could act as fast and reliable biomarkers for diagnosing and monitoring the progression of fatigue over time.

Dysautonomia presents as increased sympathetic to parasympathetic activity and is associated with fatigue in other autoimmune disorders. Increasing evidence for vagal dysregulation following COVID‐19 (Dotan et al., 2022; Pan et al., 2021) suggests that vagal underactivation may be the root cause of this dysautonomia. Recent studies found that 4–5 weeks of daily non‐invasive vagus nerve stimulation (nVNS) reduced fatigue and inflammatory markers in patients with autoimmune associated fatigue (Aranow et al., 2021; Tarn et al., 2019). This strengthens the case that vagal hypoactivity might be causal in producing fatigue and suggests nVNS might be an effective therapy for fatigue. A similar approach in those with post‐COVID fatigue is under investigation, looking into the effects of vagus nerve stimulation via the auricular branch on fatigue, and the physiological, neurophysiological, behavioural and immunological correlates of fatigue.

Although SARS‐CoV‐2 is primarily a respiratory infection, it is a multi‐system disease (Merad et al., 2022), including the nervous system. Understanding how dysfunction affects different interacting organ systems is important for understanding this disease, and discovering the abnormal subcellular alterations in function is likely to be vital for developing effective treatment strategies.

## 9 MICROCLOTS AND ENDOTHELIAL DYSFUNCTION

Potential mechanistic explanations for fatigue and other long COVID symptoms include abnormalities in tissue oxygen availability, due to vascular dysfunction and hypercoagulation, and mitochondrial dysfunction disrupting cellular bioenergetics. Coagulopathies and endothelial dysfunction are key pathologies during acute and post‐acute COVID‐19 (Levi et al., 2020; Pretorius et al., 2021; Willyard, 2020; Zuin et al., 2022), with the SARS‐CoV‐2 spike protein potentially activated by clotting factors (Kastenhuber et al., 2022). These pathologies are increased even in vaccinated individuals (Al‐Aly et al., 2022) and likely underlie the increased risk of adverse cardiovascular events in COVID‐19 survivors (Xie et al., 2022). It is therefore important to understand how endothelial dysfunction and coagulopathies arise and how they impact on tissue function.

Growing evidence also suggests that viral products, immune cells and/or inflammatory mediators may be central in the pathophysiological mechanisms that drive the persistent long COVID symptoms. Several studies show persistence of SARS‐CoV‐2 RNA several months after acute infection, though this has not yet been clearly linked to long COVID symptomology, while acute SARS‐CoV‐2 infection may dysregulate the immune system to allow reactivation of other persistent viruses (Proal & VanElzakker, 2021).

Platelets and endothelial cells may interact with viral products and circulating inflammatory molecules to induce hypercoagulation (Fogarty et al., 2021; Gavriilaki et al., 2021; Grobler et al., 2020), blocking small blood vessels and interfering with oxygen delivery. Inflammatory signalling from a dysfunctional endothelium can trigger coagulation pathways (Bonaventura et al., 2021). Antibodies from severe COVID‐19 patients can also induce procoagulant platelets and platelet apoptosis (Althaus et al., 2021). Alternatively, hypercoagulation can be triggered by SARS‐CoV‐2 itself: the SARS‐CoV‐2 spike protein, S1, activates platelets to increase inflammatory signalling, including increasing cytokine production from monocytes (Fard et al., 2021; Li et al., 2022). S1 also induces structural changes in blood‐borne molecules including the soluble plasma protein fibrinogen, increasing their aggregation and making them resistant to trypsinisation (Grobbelaar et al., 2021). Such aggregation can also be initiated by protein–protein interactions between fibrinogen and other viruses or inflammatory molecules (Kell et al., 2022).

**FIGURE 1:**
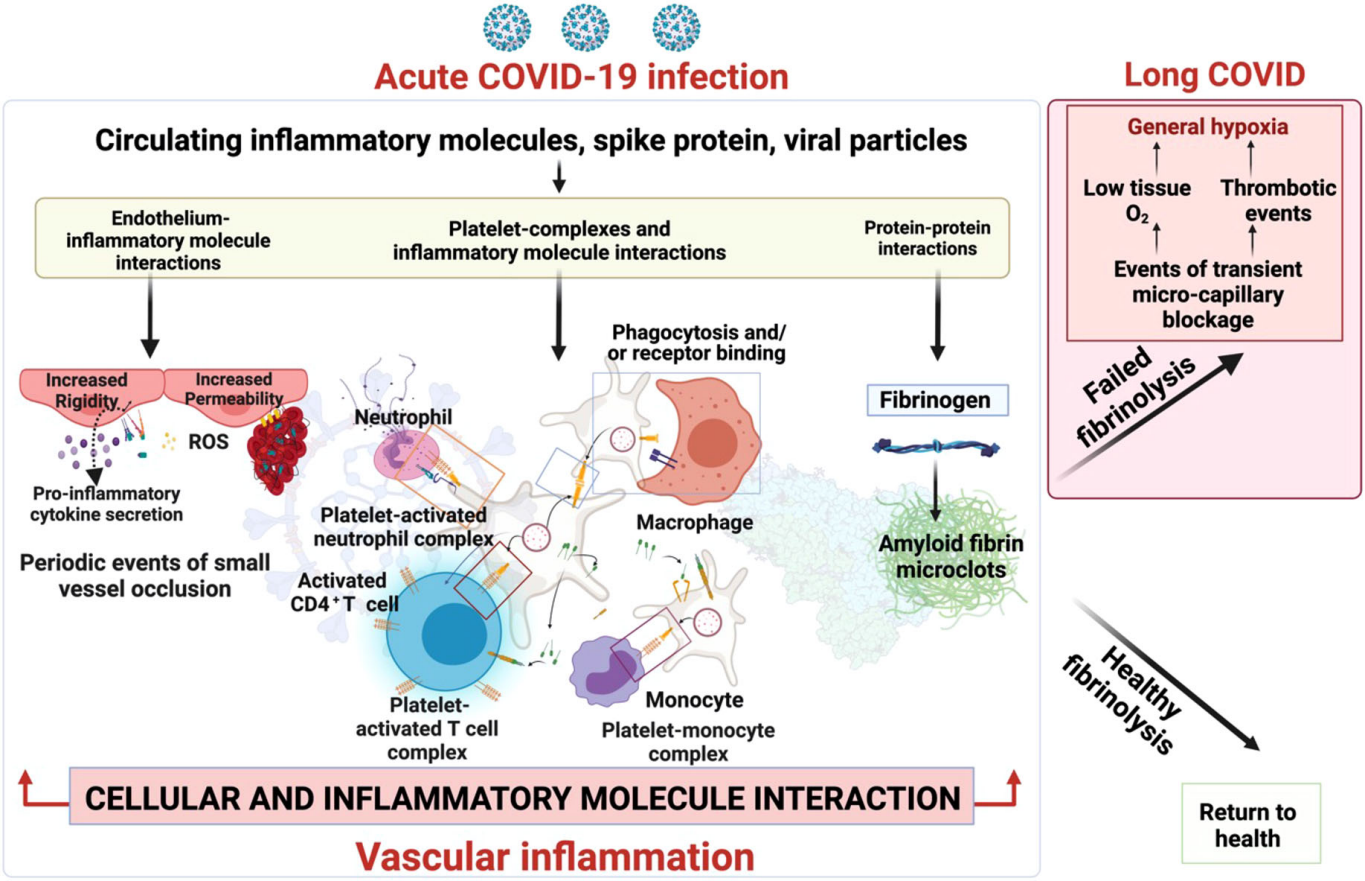
Clotting pathologies in long COVID. ROS, reactive oxygen species. Created with BioRender.com.

Microclots similar to those triggered by S1 have recently been found in the circulation of long COVID patients (Pretorius et al., 2021). These microclots are resistant to fibrinolysis, with numerous inflammatory molecules that may perpetuate both clotting pathology and systemic endothelitis found trapped inside them. These entrapped molecules include fibrinogen, von Willebrand factor, α_2_‐antiplasmin (which prevents clot breakdown via the typical fibrinolytic processes), and plasminogen activator inhibitor‐1 (Pretorius et al., 2021). The result is a failed clotting physiology, which may ultimately cause systemic tissue ischaemia and hypoxia (Figure 1).

Widespread cellular oxygen deprivation may result in many of the persistent symptoms seen in long COVID, and could account for the above‐mentioned reductions in oxygen consumption observed after CPET. Further investigation of these mechanisms, and potential interventions to combat them will be of importance for treating long COVID, as recognised by the recent report from the US Government Accountability Office (2022) noting autoimmune responses, viral persistence, organ damage and microclotting as essential areas of long COVID research.

## 10 MITOCHONDRIAL DYSFUNCTION AND FATIGUE

In addition to disrupted tissue oxygen supply, oxygen reaching a cell may not be able to generate sufficient ATP, due to aberrant mitochondrial function. Early studies have hypothesised, based on experimental observations, that that SARS‐CoV‐2 can hijack the mitochondria and exploit them for survival (Singh et al., 2020). Inflammasome activation due to mitochondrial compromise results from ineffective interferon production as a response to viral infection, increased oxidative stress and, in some, prolonged activation of the innate immune system (Moriyama et al., 2020; Singh et al., 2020). As the virus replicates in the mitochondria, their metabolic capacity may be altered, perpetuating an enhanced inflammatory response exacerbating disease severity. Experimental work carried out by Ajaz et al. (2021) and others (Gibellini et al., 2020) confirmed evidence of mitochondrial dysfunction, metabolic alterations with an increase in glycolysis and high levels of mitokines in peripheral blood mononuclear cells from COVID‐19 patients, with the latter correlating with disease severity. Host‐dependent dysregulation of glycolysis and mitochondrial metabolism was also seen in analysis of transcriptomic data from nasopharyngeal swab, peripheral blood mononuclear cells, lung biopsy and bronchoalveolar lavage from patients with COVID‐19 (Moolamalla et al., 2021).

Insights from CPET also lend support to a dysfunctional metabolism contributing to post‐COVID‐19 exercise limitation. Reduced peak oxygen consumption has been reported in several post‐COVID CPET studies, independent of cardiopulmonary limitations (Baratto et al., 2021; Cassar et al., 2021; Clavario et al., 2021; Rinaldo, Mondoni, Parazzini, Baccelli, et al., 2021; Singh et al., 2022), indicating reduced peak oxygen extraction (Singh et al., 2022), and possibly an impairment in fatty acid oxidation (de Boer et al., 2022). Whilst reduced oxygen delivery due to aberrant peripheral vasomotor response (Evers et al., 2022; Singh et al., 2022) can provide a possible explanation for these findings (secondary to autonomic and endothelial dysfunction), altered mitochondrial function also remains a possibility.

Metabolic imaging using non‐invasive techniques such as ^31^P magnetic resonance spectroscopy (^31^P‐MRS) of the skeletal muscle and heart has demonstrated abnormalities in mitochondrial respiration in closely related conditions like ME/CFS (McCully et al., 1996; Wang et al., 2021; Wong et al., 1992). Evidence of impaired oxidative phosphorylation and increased skeletal muscle pH on ^31^P‐MRS among patients with fatigue‐dominant long COVID has been observed (B. Raman et al., unpublished work), raising the possibility that similar mitochondrial dysfunction contributes to long COVID as well.

In ME/CFS patients, many aspects of mitochondrial function and metabolism appear to be altered, with reports of abnormal mitochondrial respiratory chain complex activity, pyruvate dehydrogenase activity (Brown et al., 2015; Fluge et al., 2017), complex V inefficiency (Missailidis et al., 2020; Sweetman et al., 2020), impaired neutrophil‐derived ATP profile (Myhill et al., 2009), and altered oxygen consumption of live plated cells (Tomas & Newton, 2018; Tomas et al., 2017). These data, combined with insights from comprehensive proteomic, transcriptomic (Helliwell et al., 2020) and metabolomic studies, (Armstrong et al., 2015; Fluge et al., 2017; Naviaux et al., 2016; Smits et al., 2011) lend support to an energetic explanation for symptoms among ME/CFS patients. Future research will be needed to establish whether similar alterations occur in long COVID patients, and whether metabolic therapies that restore mitochondrial function may be of benefit to patients.

Several therapies (pharmacological and non‐pharmacological) have demonstrated their potential to improve mitochondrial function in parallel disease models. These include coenzyme Q_10_ (Mizuno et al., 2008), α‐lipoic acid plus acetyl‐l‐carnitine (Logan & Wong, 2001), NADH (Castro‐Marrero et al., 2015, 2016; Forsyth et al., 1999), resveratrol (Moriya et al., 2011), methylphenidate hydrochloride (Montoya et al., 2018), *N*‐acetyl cysteine (Logan & Wong, 2001; Poe & Corn, 2020), ubiquinol, vitamin E (Logan & Wong, 2001), carefully tailored exercise rehabilitation programmes and others. There are currently numerous trials underway to evaluate the efficacy of some of these in long COVID patients (accessible on https://clinicaltrials.gov/ct2/home). A new addition to this list are endogenous metabolic modulators, a mixture of branch chain amino acids and amino acid derivatives, engineered in distinct ratios to reset multiple biological pathways, improve cellular energetics and restore homeostasis to optimise mitochondrial function. AXA‐1125 is one such example which has been shown to improve metabolic and inflammatory pathways in patients with non‐alcoholic steatohepatitis (Hamill et al., 2021). A randomised double‐blind placebo‐controlled clinical trial to evaluate the efficacy of this therapy in fatigue‐dominant long COVID is currently underway in the UK and seeks to test the hypothesis that restoration of mitochondrial metabolism will improve fatigue in patients with long COVID.

Long COVID is a multi‐system condition causing dysfunction of respiratory, cardiac and nervous tissue, at least in part likely due to alterations in cellular energy metabolism and reduced oxygen supply to tissue. Successful treatment strategies are therefore likely to directly or indirectly target these alterations.

## 11 REMOTE INSPIRATORY MUSCLE TRAINING TO TARGET LONG COVID

Many people recovering from COVID‐19 experience prolonged symptoms, including SoB, which limit activities of daily living. Indeed, the return to ‘normality’, whether through activities of daily living, work or exercise, is often associated with an exacerbation of symptoms, presenting a range of challenges for individuals recovering from COVID‐19 (Shelley et al., 2021). Given the vast number of individuals affected, there is a need to identify safe, effective and sustainable rehabilitative strategies.

Inspiratory muscle training (IMT) employs restricted airflow breathing to elicit a hypertrophic response in the respiratory muscles similar to that observed in the peripheral musculature following a strength training programme (Enright et al., 2006). IMT has been demonstrated to elicit clinically meaningful improvements in dyspnoea and quality of life in chronic obstructive pulmonary disease (COPD) (Beaumont et al., 2018) and to be well tolerated and perceived as beneficial in those with bronchiectasis (McCreery et al., 2021). As poor outcomes following COVID‐19 infection are, in part, predicted by respiratory muscle weakness (Severin et al., 2020), IMT could represent a feasible, home‐based rehabilitation method.

The aim of the IMT project was to evaluate the potential of home‐based IMT to enhance and accelerate COVID‐19 recovery. Specifically, the study assessed the influence of 8 weeks of IMT on respiratory function, SoB, exercise tolerance, daily physical activity, and perceptions of health and well‐being (McNarry et al., 2022). Participants suffering ongoing symptoms for at least 4 weeks after COVID‐19 infection were randomly assigned to either the intervention or control group, with a 4:1 weighting. IMT was associated with significant and clinically meaningful increases in health‐related quality of life, with increases across all subdomains including SoB (IMT: *n* = 111; controls: *n* = 37). The magnitude of improvement in the severity of SoB was twice the level considered clinically meaningful. IMT also improved respiratory muscle strength, estimated aerobic fitness and moderate physical activity levels, with reductions in time spent sedentary. Therefore, IMT may represent an important home‐based COVID‐19 rehabilitation strategy. Given the diverse nature of long COVID, further research is warranted on the individual rehabilitation responses. The significant withdrawal rate observed within this study (37% of those who received the intervention did not attend the post‐IMT session) highlights that no one strategy is likely to be appropriate for all. To address this variability, larger studies will be particularly valuable, tracking multiple patterns of symptoms and response sensitivities to multiple interventions, for example in integrated care pathways.

## 12 STIMULATE‐ICP

Integrated care pathways (ICP) have proven effectiveness in management of several long‐term conditions by crossing the whole pathway from primary to secondary care across the MDT (Campbell et al., 1998). The ICP approach may allow long COVID to be investigated and treated in a holistic manner, facilitating the urgently needed, scalable and generalisable solutions.

The NIHR‐funded STIMULATE‐ICP (Symptoms, Trajectory, Inequalities and Management: Understanding Long‐COVID to Address and Transform Existing Integrated Care Pathways) programme will deliver knowledge to clinicians and scientists, evidence to policymakers, and improved care to patients, while collecting real‐world data at scale (Banerjee et al., n.d.). The team spans a wide range of relevant clinical and academic disciplines including primary care and specialist services, epidemiology, mental health and health economics. Over 2 years, using the newly established 90 long COVID clinics to conduct large‐scale research, the programme has three important aspects.

First, the trajectory and healthcare utilisation of individuals referred to long COVID clinics will be studied using routine EHR, specific patient registries, and patient, health professional and policymaker interviews. These data will inform policies to reduce individual and healthcare system burden.

Second, a complex intervention trial will assess two components of a novel ICP for long COVID: Coverscan (a multi‐organ MRI scan to rule out organ impairment) and Living with COVID Recovery (a digitally enhanced rehabilitation platform). The cluster‐randomised trial will include individuals with long COVID, referred to specialist clinics in 6–10 areas (initially Hull, Derby, Leicester, Liverpool, London, Exeter) by randomising primary care networks, to allow for diversity of geography, clinical service delivery design and socio‐economic status. In addition, a nested drug platform trial will randomise individuals to drugs which could be repurposed for management of long COVID: initially rivaroxaban (an anticoagulant), colchicine (an anti‐inflammatory) and famotidine/loratadine (antihistamines), based on proposed underlying mechanisms supported by preclinical data, including coagulopathy and inflammatory cytokine production (Figure 1). Further drug arms will be added based on emerging data. The trial aims to recruit 4500 individuals over a 10–12 month period. The primary outcome is fatigue on the fatigue assessment scale at 3 months, but a wide range of secondary outcomes (physical, psychological and functional) will be collected and analysed.

Third, inequalities and stigma in long COVID care will be investigated, and an intervention developed to improve referrals from communities and groups under‐represented in current care. A mixed‐methods study is also being conducted to compare and contrast long COVID and its care with other long‐term conditions to inform future integrated care of these diseases beyond the pandemic.

Overall, the STIMULATE‐ICP has the potential to provide policy‐relevant research informed by patients, health professionals and policymakers in order to quickly establish evidence‐based, effective care for this new disease.

## 13 CONCLUSIONS

With an ever‐expanding patient population, long COVID is now a common condition with societal as well as personal impacts that necessitate a better understanding of the symptom trajectory, underlying mechanisms and treatments in order to improve population health across the globe. Much progress has been made in terms of testing paradigms that reveal the pattern of pathophysiological changes to the respiratory, autonomic and cardiovascular systems, which aid diagnosis as well as revealing the underlying functional changes in long COVID and related conditions such as ME/CFS. A number of mechanistic changes may underlie symptoms, including disruption to cellular energy production due to mitochondrial dysfunction, decreased oxygen supply due to coagulopathy and endothelial damage, and immune dysregulation. Rehabilitation can be effective, but presents a resource challenge in providing sufficient monitoring to match activity to physiological capabilities to avoid exacerbating damage. Pharmacological treatments are likely to only be effective in subpopulations of patients with specific symptoms and underlying pathology. Multi‐disciplinary approaches spanning epidemiology, immunology, multi‐system physiology and clinical research are therefore required to understand the different, interacting processes at play and to understand how to best combat them to restore health. Some of these collaborative projects are currently ongoing, but more will be needed in the future to support patients suffering from long COVID.

## CONFLICT OF INTEREST

A.B. is a Trustee of the South Asian Health Foundation and of Long COVID SOS. B.R. has received speaker honorarium fees from Axcella Therapeutics.

## AUTHOR CONTRIBUTIONS

Rónan Astin, Amitava Banerjee, Mark R. Baker, Melanie Dani, Elizabeth Ford, James H. Hull, Phang Boon Lim, Melitta McNarry, Karl Morten, Oliver O'Sullivan, Etheresia Pretorius, Betty Raman, Demetris S Soteropoulos, Maxime Taquet and Catherine N. Hall conceived, wrote and critically revised the work. All authors approved the final version of the manuscript and agree to be accountable for all aspects of the work in ensuring that questions related to the accuracy or integrity of any part of the work are appropriately investigated and resolved. All persons designated as authors qualify for authorship, and all those who qualify for authorship are listed.
